# Innovative Processing Technologies to Develop a New Segment of Functional Citrus-Based Beverages: Current and Future Trends

**DOI:** 10.3390/foods11233859

**Published:** 2022-11-29

**Authors:** Ana A. Vilas-Boas, Daniela Magalhães, Débora A. Campos, Sebastiano Porretta, Giovanna Dellapina, Giovanna Poli, Yildiray Istanbullu, Sema Demir, Ángel Martínez San Martín, Presentación García-Gómez, Reda S. Mohammed, Faten M. Ibrahim, El Sayed El Habbasha, Manuela Pintado

**Affiliations:** 1 Universidade Católica Portuguesa, CBQF—Centro de Biotecnologia e Química Fina—Laboratório Associado, Escola Superior de Biotecnologia, Rua Arquiteto Lobão Vital 172, 4200-374 Porto, Portugal; 2 Experimental Station for the Food Preserving Industry, Department of Consumer Science, Viale Tanara 31/a, I-43121 Parma, Italy; 3 Central Research Institute of Food and Feed Control, Adalet M, 1. Hürriyet Cd. No:128, 16160 Osmangazi, Bursa, Turkey; 4 National Technological Centre for the Food and Canning Industry (CTNC), C. Concordia, s/n, 30500 Molina de Segura, Murcia, Spain; 5 Pharmacognosy Department, Pharmaceutical and Drug Industries Research Institute, National Research Centre, Cairo P.O. Box 12622, Egypt; 6 Medicinal and Aromatic Plants Research Department, Pharmaceutical and Drug Industries Research Institute, National Research Centre, Cairo P.O. Box 12622, Egypt; 7 Field Crops Research Department, National Research Centre, Cairo P.O. Box 12622, Egypt

**Keywords:** functional beverages, citrus, food processing, by-products, circular economy

## Abstract

The food industries are interested in developing functional products due to their popularity within nutritional and healthy circles. Functional fruit-based beverages represent one of the fast-growing markets due to the high concentrations of bioactive compounds (BCs), which can be health promoters. Hence, functional beverages based on citrus fruits are a potential way to take advantage of their nutritional and bioactive properties that could attract the interest of consumers. In order to ensure microbial and quality stability, the beverages are subjected to preservation treatment; however, the application of high temperatures leads to the loss of thermolabile BCs. Nowadays, innovative processing technologies (IPT) such as pulsed electric field (PEF), high-pressure processing (HPP), ultrasound processing (US), ohmic heating (OH), and microwave (MW) are a promising alternative due to their efficiency and low impact on juice BCs. The available literature concerning the effects of these technologies in functional fruit-based beverages is scarce; thus, this review gathers the most relevant information about the main positive and negative aspects of the IPT in functional properties, safety, and consumer acceptance of functional citrus-based beverages, as well as the use of citrus by-products to promote the circular economy in citrus processing.

## 1. Introduction

Over the last decade, significant research attention in food fields has focused on addressing the current market demands of consumers by creating new alternatives to healthier foods. Consumers are increasingly looking for safe and natural products with generally recognised as safe (GRAS) status, created using sustainable technologies, and targeting functional properties [1,2]. One of the main consumers’ motives for purchasing functional products is related to a healthier lifestyle to mitigate chronic illness or to optimise general well-being by increasing energy and boosting the immune system [3]. This growth is determined by the consumers’ acknowledgement of the evident link between a balanced diet and health. As far as the consumers’ well-being is concerned, boosting immunity has become one of the primary focus for beverage producers and consumers [3,4]. Beverages are no longer considered simply to quench thirst, they are essential contributors to the daily intake of nutrients, and consumers look for specific functionalities to improve their well-being suitably, and beverages are by far the most accepted and common functional food due to their convenience, shelf-stability, and great matrix with desirable nutrients and bioactive compounds (BCs) [5].

In 2019, functional beverages engaged more than half of the total market of functional foods (USD 168 billion) [6], while fruit-based beverages were one of the fastest-growing segments in the global beverages industry, displaying a growth of 7.8% between 2017–2022 [7]. Citrus juice, especially orange juice, is the most consumed fruit beverage. It is associated with various nutritional health benefits, already proven by several human clinical trials, mainly related to their content of vitamins and BCs, particularly phenolic compounds [8,9,10,11]. Similar to other beverages, functional fruit beverages need to be subjected to preservation treatment before being packaged to ensure food quality and safety during the storage time [12]. Until now, juice stability was achieved by thermal processing (pasteurisation or sterilisation), performed through high temperatures to destroy microorganisms and enzymes that if present in fruit juice cause undesirable changes in odour, appearance, or taste, as well as enzymatic browning in the final product [13]. However, the application of high temperature tends to reduce the nutritional quality attributes and freshness once vitamins and phenolic compounds, which are particularly important to attribute the beverage as functional, are thermolabile compounds [14].

Aligned with the current consumer demands to provide safer and minimally processed foods with high-quality attributes, the food industry and scientific community have been encouraged to find efficient innovative processing technologies (IPT) to maintain the nutritional and sensory qualities at the same time, providing food microbiological and shelf-life stability [15]. Consequently, non-thermal and thermal methods have been suggested during the last decades, including pulsed electric field (PEF), high-pressure processing (HPP), ultrasound processing (US), ohmic heating (OH), and microwaves (MW). These emerging techniques are a promising alternative to conventional processing due to their energy efficiency and low impact on juice quality, nutritional profile, and bioactivities. Moreover, the IPT have low greenhouse gas emissions and energy consumption, resulting in lower environmental impacts; hence, they are considered sustainable technologies that align with the 2030 Agenda for sustainable development proposed by the EU directive. However, the literature available concerning the impact of these technologies on the functional properties of citrus-based beverages is scarce.

Thus, the main objective of this review is to present an overview of functional citrus-based beverage development, the use of citrus by-products to develop new functional beverages, and the main impact of IPT on functional properties, safety, and consumer acceptance.

## 2. Functional Citrus-Based Beverages

The concept of functional beverages is mostly recognised by consumers as products providing health benefits beyond their nutritional value. Moreover, consumers have become more aware of the relationship between illness prevention and a healthy diet. Consequently, the food industry continuously innovates and develops new products within this field, aiming to catch the consumers attention. However, the definition of functional foods still lacks a universal consensus [16]. The term “functional food” began to be used in the year 1930 in Japan with a milk-based fermented drink called Yakult, which claimed to keep the gut healthy [16]. Later, the Japan government defined a new product category—food for specific health uses—and created a dedicated legislative framework. They were followed by the USA, who in the 1990s developed the first claim but without providing a formal definition of functional foods. EU countries acquired the functional foods term more than 10 years later without a formal definition [17]. Although an official internationally definition of functional foods does not exist, a consensus on the main aspects has been reported in the literature, where functional foods are defined as foods that may provide health benefits beyond the fundamental nutrition values because they are rich in certain bioactive compounds [4]. Among several functional products, functional beverages are described as the most accepted and popular category, contributing to the increased consumption of healthy products worldwide [5]. According to recent reviews from Nazir et al. [3] and Corbo et al. [2] concerning functional beverages, there are four principal categories of functional beverages: (1) dairy-based beverages; (2) energy beverages; (3) sports drinks; and (4) vegetable- and fruit-based beverages [18]. Briefly, sports and energy drinks intend to prevent dehydration by providing electrolytes and carbohydrates to help athletes and, provide sustenance and improving concentration, endurance, and performance, respectively. The last category is mainly rich in green tea, ginseng, guarana, and caffeine and sometimes supplemented with some vitamins and minerals to enhance mental alertness and physical performance [19]. On the other hand, functional dairy beverages have as their main component milk or milk derivatives (yoghurt and fermented milk) and are supplemented with probiotic bacteria or other BCs, such as omega-3 fatty acids, alpha-linoleic acid, conjugated linoleic acid, and phytosterols, which are supplemented to promote nutritional health benefits [3]. Furthermore, milk-based beverages supplemented with minerals (calcium, iron, and magnesium) are already sold on the market. Recently, bioactive peptides have also been used to fortify dairy-based beverages, Evolus^®^, produced by Valio Ltd., Finland, is an example of a commercially available beverage with an added bioactive peptide synthesised by *Lactobacillus helveticus*. However, clinical data showed an inconsistent but perhaps increased risk of developing cancer with dairy consumption [20]. In addition, lactose intolerance is also one of the major concerns related to the consumption of functional dairy products. Therefore, the search for alternatives has increased in recent years. Fruit-based beverages proved to be an excellent alternative to fortified with several BCs and probiotics furthermore, fruit and vegetables are themselves rich in vitamins, minerals, and phenolic compounds, which are described as health promoters mainly by boosting the immune system. Recent studies showed that orange juice is suitable for lactobacilli supplementation and is considered ideal for consumers that are allergic to dairy products. In addition, the supplementation of orange juice with orange pomace fibres improved the flavonoid bio-accessibility [21]. Notwithstanding, fruit juice (100% fruit), nectar (25–99% fruit), and juice drink (up to 25% fruit) production represents one of the fastest-growing areas in the global beverage industry, as nearly 60% of adults consume fruit-based juices daily [22].

Within fruit-based beverages, citrus juice is an unfermented liquid extract from different commercial citrus fruit varieties produced by squeezing [23]. The most common varieties used are oranges, lemons, mandarins, grapefruit, and bergamot; however, orange juice is the most consumed fruit-based beverage worldwide due to its high nutritional value and pleasant taste (Table 1) [24]. In 2020, global orange juice consumption reached the value of 1.6 million metric tons [25]. This beverage is naturally rich in vitamin C, vitamin A, folic acid, thiamine, calcium, and potassium [8,26]. It is also rich in phenolic compounds such as flavonoids (hesperidin, neo hesperidin, and narirutin), terpenoids, essential oils, and carotenoids, which have several health-promoting benefits reported [27,28]. The high contents of vitamin C and phenolic compounds are the main contributors to the excellent total antioxidant activity of orange juice. Nevertheless, phenolic compounds have no nutritional health claims yet attributed. In addition, lemon juice is also very appreciated by all age population groups due to its refreshing taste and human health benefits. Furthermore, concentrated lemon juice showed antimicrobial potential in several works due to its high content of citric acid, vitamin c, and phenolic compounds [29]. In addition, the lemon juice is also rich in vitamin C; for instance, juice from one lemon provides approximately 18.6 milligrams of vitamin C. While the excessive sourness, bitterness, and tartness of bergamot, grapefruit, and pomelo juice have limited their consumption and commerciality [30]; however, like orange and lemon juice, they are a rich source of naturally occurring phytochemicals and vitamin C. Therefore, depending on the fruit type used, the beverage possesses different contents of these BCs and, consequently, different antioxidant capacities [22]. In this sense, it has been observed that a mix of two or more fruits can result in beverages, commonly named “smoothies”, with a higher concentration of vitamin C and BCs [22]. Additionally, the combination of fruit juices with milk or vegetable milk provides a synergistic effect as milk is rich in calcium, conjugated linoleic acid, proteins, and fat-soluble vitamins; and vegetable milk is rich in essential amino acids and minerals, as well as linoleic (omega-6) and alpha-linolenic (omega-3) acids [27,31,32]. Therefore, smoothies provide a considerable concentration of different active substances that can enhance the functional properties of the final beverage and, in some cases, improve its physicochemical characteristics, making it more attractive for consumers [22]. However, the preservation of physical stability and biological activity of the final product are important parameters to be studied when blending these two types of BCs.

Hence, orange and lemon juice, as well as other citrus-based beverages, already highly valued because of their unique nutritive composition, are a valuable and nutritional supplement to the Mediterranean diet [31].

### New Opportunities for Citrus Based-Beverages

The waste generation is one of the biggest environmental problems faced by the food industries, but it is also an economic problem since waste management implies high costs for companies [48]. The fruit and vegetable category accounts for the main waste generated during the food supply chain, and the discarded material consists mainly of kernels, seeds, peels, and pomace [49]. Examples of the most representative citrus fruit by-products include peels, pomace, water waste, and seeds [50]. In general, whatever the citrus variety, diverse BCs such as flavonoids, phenolic acids, anthocyanins, carotenoids, and vitamins (A, C and E), minerals, fatty acids, and essential oils can be isolated from these by-products [51]. Therefore, the use of by-products as sources of BCs for supplementing beverages is an interesting opportunity in the functional beverage development [5,52].

Regarding the citrus juice industry, the production involves the squeeze process, which allows the liquid to be separated from the solid matrix; therefore, the main associated by-products produced are the peels, pomace, wastewater, and seeds [53]. The quantity of by-products obtained varies depending on the source; for example, in orange juice production, approximately 50% of the total fresh orange weight is waste, the peels being the most representative by-product [54]. The high content of flavonoids in citrus peels can explain several reported biological properties, such as antioxidant, anti-inflammatory, antidiabetic, and anticarcinogenic activities [55]. In juice production, these compounds are retained within the fibre and form strong linkages that cannot be broken during the squeeze process to extract the juice; therefore, fibre ends up acting as a scavenger. Furthermore, it has been shown that this fibre also has health benefits, increasing the importance of pomace as one of the most promising sources of BCs [56]. Pectin is one of the principal components available in citrus fruit waste streams as orange juice by-products. When extracted, it has huge applications as a natural food ingredient owing to its various techno-functional properties (gelling agents, thickeners, emulsifiers, stabilisers, and texturisers). In the beverage processing, one of the main applications is the stabilisation of smoothies since the proteins from milk interact with acidic juices [57]. Ongoing works and reviews have described the beneficial effects of pectin consumption on human health, acting upon lowering the risk of diabetes, obesity, and certain gastrointestinal and inflammatory bowel diseases [58]. For instance, the supplementation of orange juice with pomace improved stool frequency in the volunteers who consumed the supplemented fruit beverage for three weeks. Furthermore, orange pomace increased the daily dietary fibre intake [59].

In Table 2, some examples of functional beverages developed with citrus by-products are summarised. Orange juice waste fraction is rich in flavonoids, phenolic acids, pectin and vitamin C [60]; hence, these orange by-products could be used as raw material to produce extracts with several bioactive properties. Recently, Adiamo et al. [60] reported the development of functional carrot juice using an orange peel polyphenol extract. Briefly, the authors conclude that adding polyphenol-enriched extracts from orange peels to carrot juice enhanced its phenolic contents and antioxidant activity. The same conclusion was observed in other studies when incorporating encapsulated polyphenolic extract from lime waste in orange juice [61]. Lemon fruit production entails a higher amount of non-compliant fruit featuring a lower appearance quality that cannot be sold in a fresh fruit market, requiring additional sustainable approaches that would contribute to the decrease in waste generation. [62]. In this sense, works from Agulló et al. [62] developed a new bioactive beverage rich in anthocyanins and flavanone with non-compliant lemons as a sustainable concept to take advantage of the non-compliant lemons that do not allow their commercialisation for fresh consumption. Another research study reported the increase in antioxidant potential and vitamin C in lime juice after the incorporation of basil and ginger by-product polyphenol extracts [63].

## 3. Beverage Processing Technologies

Pasteurisation and sterilisation are the most conventional processing technologies that guarantee the efficient reduction in microorganisms and destruction of enzymes that can cause undesirable changes [67]. The heat is generated from the energetic source and then transferred from the external to the internal part of the material by conduction and/or convection [68]. However, the application of high temperatures can degrade thermolabile compounds (vitamins and phenolic compounds) and cause undesirable changes in physical, nutritional, and sensory properties [69]. Encouraged by the consumers’ demands for minimally processed and clean-label foods, the food industry searches for new processes capable of maintaining the nutritional and sensory quality of the food, as well as microbiological stability and shelf-life [15]. The most popular methods proposed in recent years include thermal (OH and MW) and non-thermal (PEF, HPP and US) methods. Nonetheless, the latter ones are of particular interest due to their high capacity to retain nutritional and bioactive properties while maintaining the fresh-like characteristics [70] since temperature is not applied. Most of these technologies are still in the research or development stage, while some of them, for example, HPP, are already commercialised but represent only a small market. Nevertheless, these technologies are being used more widely by innovative food companies or for the development of novel food products, and their main advantages compared to conventional pasteurisation and sterilisation are: (1) shortened time processing; (2) quick heating; (3) improvement in freshness quality; (4) enhancement of bioactive properties; (5) extended shelf-life [71]. Along with HPP, MW and PEF are the most significant technologies commercially launched in the beverage industry in the last 5 years. However, PEF will remain on top with HPP, while MW will be replaced by other non-thermal technologies due to the increasing demand for beverages with high nutritional value and fresh-like characteristics [70].

### 3.1. Microwave Heating Treatment

The application of microwave heating (MH) allows better energy efficiency since the heating time to the target temperature is shorter. This leads to developing products with better nutritional and sensory quality when compared to those subjected to conventional treatment [72]. MH treatment is applied by using electromagnetic waves at a frequency of 2.45 GHz, but according to the literature, it ranges from 0.3 GHz to 300 GHz in beverage processing [24,73]. The principle of MH is the direct interaction of polar molecules, such as water, by dipole rotation and ionic conduction, generating heat more rapidly in comparison with conventional treatment [74]. The fruit beverages, due to their high-water content, absorb energy quickly, leading to the product reaching the desired temperature quickly without compromising the BCs present. However, the process parameters (power, temperature, and time) play an important role in the impact of the final beverage quality, although the effects and energy balance as well established, the industrial application of MW is still limited by the lack of know-how concerning the chemical and biochemical impact on the food matrix. Furthermore, the use of microwave energy in combination with controlled temperature and pressure allows for the high diffusion of fluids and may be applied to different matrices to extract BCs or to increase the bio-accessibility of BCs in the food matrix; thus, it could produce functional beverages [74,75].

Unlike conventional processing techniques, MH has several advantages such as reduced thermal gradient and quick heating, and it requires a shorter time and less energy to produce beverages with high quality and nutritional parameters [76]. Compared to other emerging technologies, the main disadvantage of this process is the application of temperature.

### 3.2. Ohmic Heating Treatment

Ohmic heating (OH) is a process of heating food by passing an electric current. [77]. The electrical energy applied is converted to heat that warms the food, the amount of which is directly related to the current induced by the voltage gradient in the field and the electrical conductivity of the food. Therefore, OH treatment provides rapid and uniform heating without degrading the quality food parameters because it generates heat internally [78], which is an advantage compared to MH. This kind of heating treatment reduces the possibility of a cold point, thermal damage, and nutritional losses and increases overall lethality against microorganisms in the food products. Ohmic treatment can be applied to foods with electrical conductivity between 0.01 and 10 S/m, a range including citrus juices [79,80]. The technology transfers to the industrial level requires a profound knowledge of the conductivity impact on food during OH treatment because the temperature attained depends on this factor. However, studies with orange–milk beverages report a greater degradation of BCs, such as phenolic compounds, with a longer holding time at a higher voltage than a longer holding time at a lower voltage [72]. However, this technology offers better energy efficiency, lower capital cost, shorter treatment time, and is an environment-friendly process [81].

### 3.3. Ultrasound Treatment

Ultrasound treatment (US) is an emergent non-thermal technology that uses waves with a frequency range from 20 kHz to above 10 MHz, which can be subdivided into three main regions: low-frequency, high-power ultrasound (20–100 kHz); intermediate-frequency, medium-power ultrasound (100 kHz–1 MHz); and high-frequency, low power ultrasound (1–10 MHz) for preservation. The frequency range selected for food processing depends on the requirements of the process in question [82]. The advantage of using the US involves less solvent and power consumption and a reduction in extraction time. It also increases the extract yield and uses water as a solvent, reducing the use of organic solvents, and therefore, reducing environmental impact [83]. However, to meet the FDA requirement (5-log reduction in microorganisms), a combination of sonication with mild heat treatment and/ or pressure is necessary depending on the product [84].

### 3.4. High-Pressure Processing

High-pressure processing (HPP) is considered the greatest non-thermal method used for food preservation [1]. This treatment is based on the application of high pressure (100–800 MPa, even up to 1000 MPa) evenly and quickly in the sample through a liquid phase, which is commonly water, improving the mass transfer rate, the solvent permeability in cells, and the diffusion of secondary metabolites [24]. For HPP treatment, the beverage is packed in a flexible container and transferred to the vessel where fluid is contained. Then, pressure is increased to reach the final pressure level through compressing the fluid. After a pressure-holding period, the vessel is decompressed, and the product is removed. The effectiveness of HPP to inactivate microorganisms (FDA requirement of a 5-log reduction in microorganisms in fruit juice beverage), enzymes, and bacterial spores is affected mainly by pressure, holding time, and initial temperature [13]. However, the small molecules, such as BCs and aromas, are not directly affected by the pressure. The main advantages of using HPP in functional beverages compared to traditional thermal processing are: (i) the ability to provide safe products in a fast-processing time, (ii) the maintenance of maximum fresh-like flavour and taste due to the lower temperatures, (iii) the environment-friendly quality of the technology since it requires only electric energy and does not generate new by-products. Furthermore, it is a green technology, which is in line with the SDGs number 13—climate change.

### 3.5. Pulsed Eletric Field

Pulsed electric field (PEF) is an emerging non-thermal technology for food preservation that employs an external electric field that yields reversible or irreversible electroporation in cell membranes for microbial inactivation. This technology uses short pulses of high electric fields with a duration of micro- to milliseconds and intensity in the range of 10–80 kV/cm [85] and minimum effects on product quality attributes. Compared with thermal processing, the main advantages of PEF are the non-thermal nature and low energy consumption with no waste generated [86], as well as preservation of the nutritional and sensory characteristics due to the very short time and temperatures. Until now, orange juices were among the most frequently considered foods in PEF studies, demonstrating the preservation of sensory attributes and the prolonged shelf-life [87]. However, the efficiency of PEF pre-treatment differs on some factors, such as extraction time, treatment temperature, pulse frequency, pulse shape, specific energy input, electric field strength, pH, pulse width, food matrix density and size, and chemical properties of extracting by-product [88].

## 4. Impact of IPT on Bioactive Compounds

The preservation of the nutritional, biological, and organoleptic properties of food is a key goal to food industry [89]. As a result, optimising preservation treatment is a crucial tool to maintain an equilibrium between the safety and nutritional quality of the new functional product. Many alternative approaches to conventional thermal treatments have been suggested for their application in various processes within citrus-based beverages to avoid a deterioration in the nutritional and biological quality of the processed product [90,91]. The most common way to consume citrus fruits and contribute to a healthy diet and healthy lifestyle is through beverages such as juices, blends, smoothies, and fermented and fortified beverages [89]. Therefore, several research studies have suggested IPT as an alternative to thermal treatments for beverage production to avoid a deterioration of the nutritional and bioactive compounds of the processed product [90,91,92].

### 4.1. Phenolic Compounds

Citrus fruit is a major source of phenolic compounds in the human diet, which include flavonoids (hesperidin, narirutin, diosmetin, diosmin, naringenin, neohesperidin, quercetin, tangeritin, and nobiletin) and phenolic acids (ferulic acid, caffeic acid, vannilic acid, and p-coumaric acid) [55,93,94]. The consumption of these BCs shows various bioactivities, such as anti-microbial, antioxidant, anticancer and anti-inflammatory, antimutagenic, and antiallergic properties [95]. In particular, two polymethoxylated flavones (tangeretin and nobiletin) present in citrus fruit and, consequently, present in juices, are of interest due to their demonstrated pharmacological potential to inhibit cancer cell growth in vitro and in vivo [96,97]. Recently, other phenolic compounds present in citrus at high quantities, diosmetin and quercetin, were identified as potential therapeutic agents for the coronavirus disease that emerged in 2019 (COVID-19) [98,99]. Furthermore, hesperidin, the main phenolic compound in orange juice (28.9 mg/100 mL juice) [100] showed a potential therapeutic effect against COVID-19 [101], currently in human clinical trials. For instance, hesperidin combined with diosmin is already used commercially to treat blood vessel conditions such as haemorrhoids, varicose veins, and poor circulation (venous stasis) [102]. For all the bioactivities these compounds demonstrate, there is a growing interest in limiting the phenolic losses during food processing as the polyphenols are vulnerable to heat, light, and other physical and chemical treatments [103]. 

Thus, several studies focus on the effects of IPT on the polyphenol content of citrus juices. Table 3 reports the beverages produced and experimental conditions, as well as the main conclusion observed after the processing. For instance, Igual et al. [104] reported an increase of 82% in total phenolic content bio-accessibility when grapefruit juice was treated by MH (90 W, 30 s) compared to conventional heating (CH). On the other hand, Bhat et al. [105] reported that most of the BCs in lime juice samples were enhanced when treated by US (25 kHz, 20 °C) for 60 min compared to samples treated for 30 min and control samples (untreated). Aadil et al. [106] also showed interesting results when they treated grapefruit juice with US (28 kHz, 20 °C) and saw a significant improvement in total phenolics, flavonoids and flavonols in all the juice samples sonicated for 30, 60, and 90 min.

Usually, the research studies compare one emerging technology with the CH; however, some studies compare the effects of two different IPT, such as the HPP and PEF methodologies, on orange juice. Although some results are contradictory, in general, HPP and PEF methodologies allowed an increase in phenolic compounds compared with pasteurisation treatment and compared with thermal IPT (ohmic and microwave heating); however, some studies demonstrated losses during the storage time for both treatments. The application of US, despite preserving the phenolic compounds in citrus juices because it does not use high temperatures, requires a much longer treatment time than HPP and PEF.

### 4.2. Vitamin C

Citrus juice is an important source of vitamin C, also known L-ascorbic acid (L-AA), a nutrient that, apart from its vitamin action, is valuable for its antioxidant effect, stimulation of the immune system, and other health benefits [94]. Vitamin C is the most important water-soluble nutrient and is related to the antioxidant capacity of citrus juices. However, it is an unstable compound and decomposes easily under less desirable conditions. Vitamin C’s degradation precedes aerobic and anaerobic pathways and depends upon many factors: oxygen, heat, light, storage temperature, and storage time. It was reported that several decomposition reactive products occurred via the degradation of vitamin C and these compounds may combine with amino acids, thus, resulting in the formation of brown pigments. Therefore, the nutritional quality of food during storage has become an increasingly important issue, and the loss of this vitamin can be a critical factor in the shelf-life of citrus juices [110]. The impact of non-thermal processing on vitamin bio-accessibility has scarcely been studied, and most of the studies are focused on vitamin C content in fruit beverages. Vitamin C is characterised by photosensitivity; therefore, it tends to oxidise quickly. Consequently, each process using elevated temperature causes loss of this vitamin compared to fresh material, which represents losses from 20% to even 90% depending on the temperature level, the duration of the processing operation, and contact with oxygen [111].

Table 4 compiles some recent studies focused on the effects on vitamin C content after processing citrus juices with different IPT. The MH technology did not show any increase in vitamin C content; on the contrary, for example, Vikram et al. [112] show degradation of vitamin C in the orange juice during MH treatment (455 W, 180 s, uncontrolled temperature) compared to CH treatment. The MW degradation of vitamin C can be attributed to the uncontrolled temperatures generated during the processing, which exceeded 100 °C and caused degradation due to the thermolabile nature of the vitamin C. On the other hand, US technology shows interesting results with an increase in vitamin C content when compared to the heat treatments or control. Bhat et al. [105] claim that this increase is attributed to the elimination of dissolved oxygen, which is essential for the degradation of ascorbic acid during the cavitation produced during sonication treatments. Some authors have compared the difference between HPP and PEF methodologies on orange juice, and contradictory results were found. For instance, Sánchez-Moreno et al. [107] showed losses for both treatments, but both treatments allowed for increase in vitamin C compared with pasteurisation/ heat treatment, and Esteve and Frigola [94] showed that orange juice treated with both PEF and HPP methodologies had a good result for vitamin C content compared with pasteurised juice.

### 4.3. Carotenoids

Carotenoids are the pigments responsible for the external and internal colouration of citrus fruits and provide a wide range of colours from yellow to red. Their contents and profiles are important indexes for the commercial and nutritional quality of the fruits and also their juices [115]. There is a strong relationship between the intake of carotenoids and health benefits, as α-carotene and β-carotene possess a special role of being a precursor of vitamin A, which is essential to human health systems [116]. Carotenoid compounds are divided into two main groups based on their structures: carotenes or pure hydrocarbon carotenoids as α-carotene, β-carotene, and lycopene and oxygenated carotenoids (xanthophylls) as antheraxanthin, lutein, neoxanthin, violaxanthin, and zeaxanthin [117]. The study of carotenoids in beverages has grown in interest due to their health benefits. However, the preservation process of citrus juices involves the use of conventional thermal pasteurisation, which is essential to prolong its shelf-life by inactivating microorganisms and enzymes but, on the other hand, accelerates the oxidation and degradation of carotenoids [87]. The decrease in carotenoid content in the final product leads to loss of bioactivity, which invalidates the final product’s functional properties. Several studies focussing on the effects of the different IPT on carotenoid content in citrus juice are summarised in Table 5. Briefly, studies from Fratianni et al. [118] reported a decrease of approximately 50% for almost all carotenoids in orange juice after 1 min of heating with MH (3 kW; 2.45 GHz). This phenomenon was associated with the high sensitivity of the bioactive compounds to conventional thermal processes. Although MH technology is not a conventional technique, it also has the disadvantage of being a heat treatment, leading to the degradation of carotenoids. Studies from Sánchez-Moreno et al. [107], comparing the main effects of HPP and PEF on orange juice, reported that the use of HPP (400 MPa/40 °C/1 min) showed better results when compared to PEF (750 µs, 35 kV/cm). Furthermore, Esteve and Frigola [94] showed that HPP (4000 bars for 5 min) and PEF (100 µs, 30 kV/cm) had good results when compared to conventional pasteurised juice, but the differences between these two techniques were observed depending on the temperature to which these techniques were subjected. A better recovery of total carotenoids was obtained with the HPP technique at a temperature = 4 ± 2 °C: 997.2 µg/100 g compared to PEF: 964.2 µg/100 g. However, for a temperature = 10 ± 2 °C, the scenario has changed, being the PEF technique with better results than HPP. These authors showed that the total carotenoid concentration decreased in the pasteurised juice (−12.8%), in the juice treated with PEF (−6.7%), and in the juice treated with HPP (−4.2%) in comparison with the fresh juice. A possible explanation for the better recovery with HPP is that carotenoids do not exist freely in the medium but rather form bonds with proteins in the cell membrane, and treatment at 4000 bars caused pressure which induced denaturation of carotene–protein bonds. Therefore, it was hypothesised that HHP may be a suitable treatment for increasing carotene extraction from the matrix and may be associated with increased nutritional value. Plaza et al. [108] showed that PEF juices (750 µs, 35 kV/cm) did not change the carotenoid content compared to freshly squeezed orange juice. However, HPP (400 mPa, 40 °C, 1 min) showed an increase in the extraction capacity of each individual carotenoid concerning the untreated juice and the total carotenoid content (45.19%). Thus, according to the studies presented in Table 5, the most suitable green technology to recover and preserve carotenoids was HPP.

## 5. Impact of IPT on Microbial Contamination, Shelf-Life, and Sensory Parameters

Thermal treatment has long been used as a processing method to extend the shelf-life of food products by eliminating or reducing spoilage and pathogenic microorganisms and enzymes. Apart from thermal pasteurisation or sterilisation, some chemical preservatives are also widely used to prevent the growth of bacteria, yeast, and moulds and increase the shelf-life of fruit juices [122]. The most used are potassium sorbate and sodium benzoate. In addition, other acids such as citric, malic, and phosphoric are generally added to achieve the acid flavour or tartness and maintain the beverage shelf-life. However, consumer demand for natural-origin, safe, and environment-friendly food preservatives or technologies has increased [4]. Natural antimicrobial agents such as organic acids, essential oils, and phenolic compounds have shown considerable potential for use in the beverage industry to avoid synthetic compounds and decrease the need for heat treatment [123]. From the scientific literature, it is apparent that a combination of IPT and natural antimicrobial agents is effective to inactivate microorganisms or reduce the log colony forming units (CFU) while not adversely affecting the sensory and nutritional quality [122,124]. However, the biggest challenge is the use of technologies that prolong the shelf-life while maintaining all nutritional and bioactive value without adding preservatives.

In Table 6 studies are reported evaluating the effect of different IPT on the shelf-life of several citrus beverages regarding microbial contamination. As previously mentioned, PEF is one of the emergent non-thermal technologies with promising results in the field of fluid foods; therefore, several research studies evaluate the impact of PEF in citrus beverages. The study by Elez-Martinez et al. [125] evaluated the effects of PEF (35 KV/cm, 4 μs pulse width, 32.5 °C) on the microbial and quality-related parameters (vitamin C, colour, and antioxidant activity) in orange juice. The results demonstrated that PEF processing could produce stable orange juice with a shelf-life comparable to that achieved with thermal processing (90 °C for 1 min). At the same time, it retained higher nutritional value during storage time (56 days of storage at 4 °C). This IPT inactivates microbial growth by induction and alterations in the transmembrane potential value [126]. Moreover, it easily adjusts the values for microorganism inactivation by inducing irreversible electroporation. In addition, orange juice treated with PEF retained better colour than heat-pasteurised juice throughout the storage time. However, no significant differences (*p* < 0.05) between treatments in pH, acidity, and °BRIX were found. Furthermore, vitamin C retention was outstandingly higher in orange juice processed with PEF, fitting the recommended daily intake standards during storage. This could be explained by the low processing temperatures (<40 °C) used in PEF treatment.

Another important non-thermal technology, mainly at the industrial level, is the HPP, which allows for the processing of foods at temperatures below those used during thermal pasteurisation or sterilisation so that the flavours and essential nutrients undergo minimal changes. Different authors showed a microbial reduction using this technology (Table 6). For instance, Wang et al. [131] reported a 5-log reduction in *E. coli* (200 MPa, 15 min, 22 °C) and 5-log reduction in *Lb. plantarum* (300 MPa, 15 min, 22 °C) in orange juice. Another interesting investigation was performed by Timmermans et al. [133], who, with the same technology using 600 MPa for just 1 min at low temperature (17 °C), demonstrated the complete inactivation of total psychrophiles, *Enterobacteriaceae*, *E. coli*, lactic acid bacteria, yeasts, and moulds. On the other hand, US processing under different processing conditions was investigated concerning the inactivation and potential subsequent growth of microorganisms in orange juice. Research studies from Valero et al. [127] reported a lower level of microbial inactivation (≤1.08 log CFU/mL with the application of ultrasonic treatment (500 kHz/240 W; 15 min). Microbial growth was observed in the substrate following 14 days of storage at refrigeration (5 °C) and mild abusive (12 °C) temperatures. The resistance of microorganisms to US increased with the presence of pulps in juice. After continuous US treatments at flow rates of 3000 L/h, negligible reductions in microbial counts were obtained. No ultrasound-related detrimental effects on the quality attributes of juice, such as colour and limonene content, were found. Nevertheless, to prevent food-borne pathogens in juice, it will be necessary to combine ultrasound treatment with other processing methods with greater anti-microbial potency to achieve a shallow initial concentration of bacteria, yeast, and moulds in the juice. Such combinations will require further exploration of significant synergistic effects relevant to industrial use.

Regarding the results presented in Table 6, it is possible to conclude that innovative processing technologies (PEF, HPP, and US) can reduce the growth of microorganisms. However, it was possible to observe differences between these non-thermal technologies. For instance, the US technology needs a more extended time to achieve a better result. In contrast, HPP technology can inhibit or even inactivate the growth of microorganisms in a short period.

## 6. The Consumers’ Perspective

**Figure 1:**
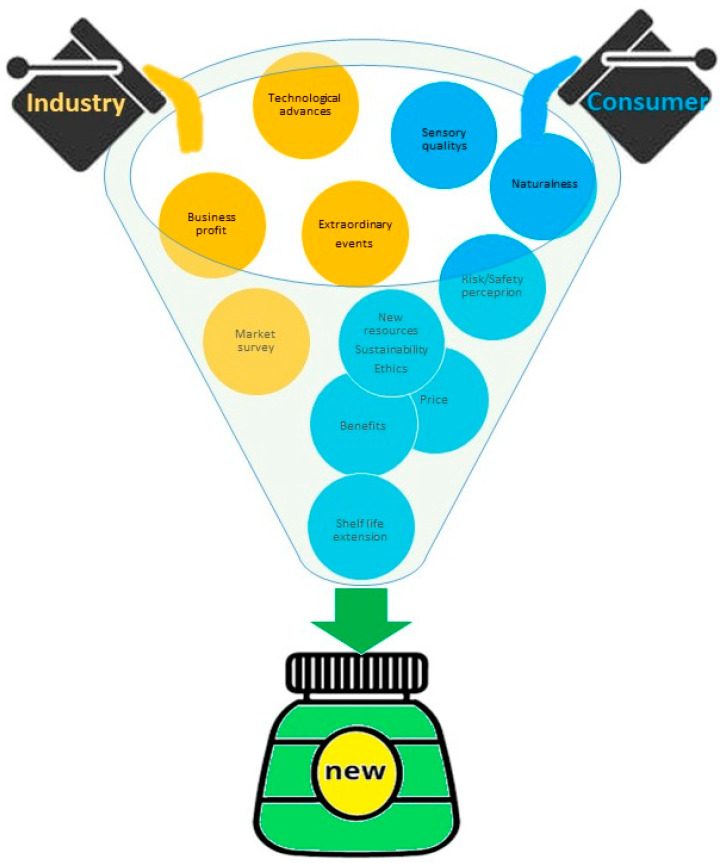
The food product development (NPD) from the perspective of the consumers (blue) and the industry (yellow).

The research on innovative and emerging food technologies has been frequently focussed on validating consumers’ acceptance of products obtained through these technologies, since their benefits are not fully perceptible by the general consumer due to low literacy in these topics. Therefore, the factors responsible for consumer choice, acceptance, and buying behaviour of beverages produced by emerging technologies are established even before their launch on the market [136]. Furthermore, functional beverages or functional ingredients to be added to the beverages are less familiar to consumers; therefore, little is known about the consumer acceptability [137]. As for food products, the sensory quality approval of functional beverages is critical to their success in the marketplace. However, on its own, the sensory quality will not guarantee success. Consumer perceptions about the price, food security, and the health benefits associated with beverages produced by IPT or the aroma and flavour associated with some functional ingredients can negatively influence consumer choice and purchase decisions. For instance, generally, the products supplemented with flavonoids, terpenoids and isoflavones are characterised by the consumers as “bitter”, “acid”, and “astringent” [137]. On the other hand, a sensory evaluation of orange juice fortified with probiotics (*Lactobacillus casei* 01 encapsulated in low-molecular-weight chitosan) with 400 consumers from Bangkok and respective suburbs concluded that 82.3% of the consumers accepted the orange juice containing probiotics beads due to its taste (9.6) and nutritional value (8.9), giving scores of texture and overall preferences as 6.6 and 6.7, respectively [138]. However, the addition of probiotic capsules negatively impacted swallowing and presented with remaining particles in the final product that were perceptible to the consumers. The data collected in NPD with direct consumer involvement can positively impact the development and enhance the company’s revenues. (Figure 1). The study of consumer behaviour and attitudes towards new food encompasses various factors, such as preference, functional attributes, sensory attributes, desire to eat, buying intentions (e.g., health motivation), product format, and frequency of consumption [6,139]. In addition, culture/geography has an important impact on the acceptance of functional beverages. Research studies with western consumers have reported that German consumer preferences depend on their own ideologies, i.e., sceptics prefer non-functional beverages, because they do not believe in the health benefits of functional foods. Furthermore, it was reported that while most UK consumers preferred the sensory characteristics of conventional orange juices, a slight segment of the population preferred the sensory attributes of functional orange juices [137]. Thus, consumers’ acceptance and purchase intentions for functional beverages were heterogeneous.

The health benefits are the main feature that distinguish functional beverages from conventional ones, and most studies have focused on consumers’ perceptions on the impact of health-related claims on consumer acceptability and purchase intentions or the effect of exposing health information on consumers’ perceived value [6]. Some studies reported that publishing the product’s health benefits to consumers positively influenced consumer acceptance and purchase intention [140,141,142]. Therefore, the food and nutrition policies and marketing product should develop products based on the target consumers to improve consumer motivation to understand the health claims by increasing their interest in healthy eating habits [143]. A health claim of a product states what the product does in terms of health, well-being, and performance and has become a way to communicate this information to consumers [3]. They significantly influence consumers’ purchasing decisions and help them make more informed choices, specifically if the claims for the beneficial actions are obvious. Another factor related to the product acceptance is the price. Based on a literature review, considerable evidence shows that the price–benefit ratio is the main determinant for acceptance and purchase of products with functional ingredients and/or processed by IPT [144]. However, even when a product provides additional health benefits, the technology that is used to produce functional food influences the acceptance of the product, since consumers may perceive new food technologies as riskier than traditional food technologies. This feeling of food risk is inversely related to the willingness to purchase the product. Hence, the marketing of products always associates them with nature, as that is a concept that is positively valued by consumers. In line with this, the results of some studies suggest that the dichotomy between new technologies and nature creates a better understanding of the acceptance of IPT by consumers.

Currently, consumers are more focused on healthy and nutritional beverages mainly from natural sources and without additives. Consumers are opting for natural plant-based beverages, as they are thought to be more beneficial than other beverages, including carbonated drinks. Furthermore, even some unpredictable events such as the pandemic can act as a driver in consumer choices. The food system was strongly affected by the COVID-19 pandemic, from production problems to shipping to retailers. Some food sectors, mainly fresh fruits and, particularly, citrus, have had a remarkable surge in consumption. The demand for fresh citrus fruits and juices has sensibly grown, mainly due to the choices made by health-conscious consumers. Orange juice was one of the leading products consumers have sought during the pandemic, with a sales increase of 22% compared with previous years. This is mainly since consumers are daily confronted with the preventive effects of a diet rich in fruit and vegetables on diseases such as COVID-19. Citrus fruit and juices are rich in flavonoids, which are highly recommended to block the coronavirus. Moreover, some of these citrus bioflavonoids revealed a potential binding with the ACE2 protein, which could prevent COVID-19 infection. Additionally, citrus fruits/beverages boost the immune system as they are also rich in vitamin C and minerals. Therefore, the new development of citrus products has the chance to create a strong relationship in consumer perception between citrus product consumption and the health benefits, which has been proven to be a critical factor in consumers choices.

## 7. Trends and Innovations

The emerging trends associated with beverage processing technologies focus on improving the process and the product sustainability in order to overcome the current environmental problems [145]. The growing consumer preference for eco-friendly products, along with new governmental directives, is pushing sustainability into the forefront position of beverage market trends. As a result, the industries have developed solutions to decrease the carbon footprint of beverage production through energy efficient technologies [146]. For instance, last year, several companies start producing fruit juice with HPP technology to preserve all its nutrients, vitamins, antioxidants, and its flavour while killing 99.999% of the microorganisms [147] and maintaining the fresh juice characteristics. Moreover, beverage companies changed their packaging material to sustainable materials, for example, plastic-free or reusable packaging, which enables them to significantly reduce the ecological impact of single-use packaging, one of the major contributors to land and marine plastic pollution [148].

On the other hand, market trends are shifting away from chemical additive use since consumers are becoming increasingly interested in organic and natural ingredients to produce products, and market trends are shifting away from synthetic products [3]. Various studies over the last decade have attempted to develop natural products with enhanced health benefits to incorporate in the food industry. Exploring the potential of functional ingredients from the food by-products is an interesting research area in producing novel functional beverages [149]. Natural ingredients with strong antioxidant activities could be used to formulate novel functional beverages; for instance, polyphenols have gained attention due to their therapeutic potential against cardiovascular diseases, obesity, type-2 diabetes, neurodegenerative diseases, and specific cancers [150,151]. On the other side, the addition of functional ingredients into beverages can lead to interactions among other compounds present, resulting in insolubility, oxidation, precipitation, or degradation of the ingredients; thus, scientific knowledge and industrial expertise are required to mitigate potential issues with the final product. For example, milk has been reported to negatively affect the bio-accessibility and bioavailability of flavonoids present in citrus juices [152]. Therefore, it is recommended not to fortify citrus smoothies with flavonoids since they lead to insolubility formation. However, the application of correct preservation treatments or the addition of food additives such as pectin can solve the problem. In addition, the amount of the ingredient used in the final formulation could not create these undesirable interactions. The optimal BCs dosage is defined as the dosage that is high enough to produce a positive effect without negatively affecting other functional and technological components present. While formulating functional beverages, the most challenging task is to ensure that the functional ingredients will remain intact, active, and are bioavailable after processing and storage [153].

The addition of antimicrobial compounds in functional fruit-based beverages is an important area that remains unexplored. Natural antimicrobials include bacteriocins, oligosaccharides and polysaccharides, essential oils, and plant extracts rich in phenolic compounds suitable for replacing the synthetic fungicides and preservatives in food products, including beverages [2,154]. In addition to the antimicrobial effects, these compounds showed other several health benefits, which draw attention from the food industry. Carotenoids and phenolic compounds, for example, obtained from fruit by-products, have the potential to be used as natural antimicrobials in beverages. They delay the rancidity and formation of off-flavour compounds, extending the shelf-life of foods and beverages. In addition to the preservative effect, they also carry numerous health-promoting properties, e.g., antioxidant activity.

Although probiotics are usually added to milk-based beverages, recent studies show positive results from their incorporation in fruit juices, namely, citrus fruit. Furthermore, food industries are looking for alternatives to milk-based beverage production due to the increased amount of lactose intolerance and the data relating milk consumption to cancer. Consequently, the supplementation of functional fruit beverages is an attractive alternative for vegans, lactose intolerant, and milk-allergic individuals [155,156]. However, several problems could occur related to the bacteria metabolisation in some beverage compounds. For example, when strains of Lactobacillus are added to orange juice containing citric or malic acid, they metabolise the acids and produce acetic acid, lactic acid, and carbon dioxide gas as by-products, which can degrade the final product by altering its normal characteristics [157]. On the other hand, the combination of probiotics with phenolic compounds represents a strong market potential in functional beverages. However, due to their inherent antioxidant properties, their sensitivity to oxygen is the major challenge with manufacturing and storing fruits as functional beverages. Natural food-based products are also being explored to address this problem. For instance, the study by Gonzalez-Molina et al. [158] demonstrated that the addition of red fruit concentrates to lemon juice protected the antioxidant properties of vitamin C and maintained the beneficial effects of the product throughout the shelf-life. Nonetheless, non-thermal processing technologies could be an excellent possibility to produce shelf-stable products while preserving the antioxidant potential of these products, as revised in this work.

## 8. Conclusions

Currently, the beverage industry is facing several challenges regarding new product developments as a response to consumers’ needs in purchasing differentiated products with high nutritional and bioactive values and clean labels, as well as being safe. The consumption of fruit juice is an essential food commodity within the global market, as well as a strategy to use surplus or overripe fruits in production. The citrus-based beverages are one of the most appreciated by consumers not only for the typical flavour but also because of the associated well-being claims as a health promoter, which are directly linked with the micronutrients and BCs present in these fruits. Therefore, the preservation of such BCs represents a great technological challenge while guaranteeing microbial safety and convenient shelf-life. As described throughout this review, the traditional thermal process (pasteurisation or sterilisation) represents a series of limitations in maintaining the nutritional value of these products. Because of consumer preference for functional processed foods with fresh-like characteristics, innovative processing technologies such as PEF, HPP, US, and MW could be a promising alternative due to the potential to safely preserve the natural value of foods over extended periods while satisfying the microbial safety and sensory quality. Despite the different technologies, HPP seems to be the technology that retains the most nutritional and bioactive qualities while maintaining shelf-life. However, the application of several novel processing technologies has been explored in orange juices but is scarce in other citrus juices, which opens opportunities for the future. The application of non-thermal technologies is of particular interest since the preservation of colour and flavour characteristics in these products has been proven.

## Figures and Tables

**Table 1:** Main nutritional and bioactivity parameters of different citrus juices: lemon, orange, grapefruit, mandarins, bergamot, and pomelo.

Parameters			Lemon Juice		Orange Juice	Grapefruit Juice Mandarin Juice	Bergamot Juice Pomelo Pink
pH		2.5–3.0	3.8–3.9	3.0–3.7	3.6–4.4	2.2–2.9	3.67
Sugars	Total content	--	84 g/L	49.8–85.4 g/L	--	--	76.5–91.0 g/L
	Glucose	7.5–7.9 g/L	25.79–29.5 g/L	22.1–24.6 g/L	14.6 g/L	9–13.1 g/L	13.1 g/L
	Fructose	5.4–5.8 g/L	22.93–25.3 g/L	22.3–26.5 g/L	17.1 g/L	9.7–12.6 g/L	13.4 g/L
	Sucrose		34.8–67.4 g/L	29.5–35 g/L	57.1 g/L	16.8–18 g/L	31.3–49.1 g/L
Organic Acids	Citric acid	3.2–44.6 g/L		16.3–23.9 g/L	--	1.6 g/L	14.15 g/L
	Malic acid	3.5–4.4 g/L		0.4–3.0 g/L	--	--	0.8 g/L
	Succinic acid	5.7–6.6 g/L		0.2–0.61 g/L	--	--	0.1 g/L
	Ascorbic acid	0.6 g/L	0.1 g/L	0.2–0.7 g/L	0.1–0.5 g/L	0.4–0.9 g/L	0.2 g/L
Phenolic Compounds	Hesperidin	81.5–117.4 mg/L	36.3–435 mg/L	3.77–100.25 mg/L	367–873 mg/L		--
	Narirutin		32.8–36.0 mg/L	37.07–120.06 mg/L	77–1450 mg/L	18–182 mg/L	13.8 mg/L
	Eriocitrin	16.7–391 mg/L	1.1–6.7 mg/L		0.1–1 mg/L	13.4–15.6 mg/L	
	Neoeriocitrin	9.7–14.3 mg/L				103–402 mg/L	16.0 mg/L
	Neohesperidin	0.9–1.6 mg/L	0.7–5.9 mg/L	0.2–24.2 mg/L	--	66–554.5 mg/L	64.8 mg/L
	Poncirin			14.2–26.0 mg/L		--	--
	Didymin	0.1–15 mg/L	8–12 mg/L	2.7–3.3 mg/L	56.8 mg/L	--	--
	Naringin	2.8–4.1 mg/L		166–464.1 mg/L	--	97–528.2 mg/L	600–1763.71 mg/L
	Naringenin	--	0.1–0.3 mg/L	24.3–31.3 mg/L	--	--	--
	Rutin	2.3–3.5 mg/L	--	12.80–14.71 mg/L	--	--	--
	Quercetin	1.5–2.6 mg/L	--	--	--	--	--
	Ferulic acid	0.1–0.2 mg/L	0.03 mg/L	14.1–26.5 mg/L	--	--	--
	Caffeic acid	3.4–4.7 mg/L	0.02 mg/L	--	--	--	--
	p-coumaric acid	0.1 mg/L	0.04 mg/L	13.7–16.30 mg/L	--	--	--
	o-coumaric acid	0.9–1.4 mg/L	--		--	--	--
	Vannilic acid	--	--	3.6–5.3 mg/L	--	--	--
	Gallic acid	--	--	3.18–4.62 mg/L	--	--	--
	Protocatechuic acid	--	0.18 mg/L	1.87–3.70 mg/L	--	--	--
	Chlorogenic acid	9.0–13.4 mg/L			--	--	--
Vitamins	Vitamin C	508–625.4 mg/L	66.6–41.0 mg/L	455–680 mg/L	250 mg/L	341–867 mg/L	--
Minerals	Potassium	1300 mg/L	1000 mg/L	74 mg/L	1200 mg/L	1359 mg/L	457 mg/L
	Iron	4.5 mg/L	0.4 mg/100 mL	--	0.5–0.6 mg/L	--	--
	Phosphorous	100 mg/L	50–210 mg/L	220 mg/L	--	--	120 mg/L
	Magnesium	70 mg/L	90 mg/L	25.6 mg/100 mL	120–133 mg/L	92 mg/L	289 mg/L
	Zinc	--	1 mg/L	--	0.3–0.4 mg/L	--	--
	Sodium	20 mg/L	5–30 mg/L	8.0 mg/100 mL	4.4–6.5 mg/L	17 mg/L	133 mg/L
	Calcium	70 mg/L	110 mg/L	29.0 mg/100 mL	39.8–43.80 mg/L	76 mg/L	165 mg/L
References		[27,32,33,34]	[27,35,36,37,38]	[27,36,39,40,41,42]	[27,43]	[27,44,45,46]	[27,36,47]

**Table 2:** Examples of functional citrus-based beverages with citrus by-products.

Product	Citrus by-Product	Target Function	Ref.
Carrot juice with orange peel extract	Orange peels	Increase the polyphenol content of carrot juice with orange peel extract.	[60]
Citrus mix juice with maqui-berry	Second Quality Citrus Fruits (orange, lemons, mandarin)	Valorisation of non-compliant citrus fruit for the development of new beverages, rich in anthocyanins and flavanones.	[62]
Functional Lemonade	Non-conforming lemons during harvest time	Develop a lemonade enriched with natural herb extracts (ginger, linden, and mint).	[64]
Orange juice with polyphenol extract from lime waste	Lime waste (Citrus latifolia)	Enrich orange juice with hesperidin from lime waste to enhance nutritional value and protect the polyphenols’ oxidation against the pasteurisation process.	[61]
Beverage with orange pomace addition	Orange pomace	Valorise the orange by-product by creating a functional orange juice that increases stool frequency in healthy adults.	[59]
Functional juice based on citrus	Citrus × clementina Hort. by-products	Ascribe antioxidant, hypoglycaemic, and hypolipidemic effects.	[65]
Orange juice with orange peels essential oil (EO)	Orange peels from different varieties	Prolong shelf-life of orange juice through anti-microbial activity of EO in decreasing Escherichia coli O157:H7 in. Sensory characteristics of juice with EO encapsulated in chitosan were acceptable.	[66]

**Table 3:** Impacts of innovative processing technologies on citrus juice phenolic compounds.

IPT	Application Matrix	Detected Compounds	Conditions	Results	Ref.
MH	Grapefruit juice	Total phenolic compounds (TPC)	900 W, 30 s	↑ Better preservation of TPC (82%) than heat treatment	[104]
MH	Orange juice–milk beverage	Total phenolic compounds	2450 MHz, 65 °C, 60 s	↑ Levels of total phenolic compounds compared to the conventional treatment (75 °C, 15 s) ↑ The MH juice showed higher ACE inhibitory activity and antioxidant activity	[72]
US	Lime Juice	TPC and flavonoids	25 kHz, 20 °C, 30 to 60 min	↑ Increase in total phenolics (263.8 up to 272.0 and 336.0 mg GAE/g), flavonoids (0.26 up to 0.30 and 0.37 mg CE/g)	[105]
US	Grapefruit Juice	Total phenols, flavonoids, and flavonols	28 kHz, 20 °C	↑ US for 90 min showed an increase in total phenols (826.27 µg/g) compared to control 0 min (757.96 µg/g), total flavonoids (603.18 µg/g) compared to control 0 min (462.27 µg/g), and total flavonols (2.94 µg/g compared to control 0 min 2.70 µg/g)	[106]
HPP	Orange Juice	TPC	400 MPa, 5 min	↑ Treated juice with HHP had a good result: (T = 4 ± 2 °C, for 7 weeks): 1.051 mg/mL compared to pasteurised juice (1.070 mg/mL); (T = 10 ± 2 °C, for 6 weeks): 1.002 mg/mL compared to pasteurised juice (0.985 mg/mL)	[94]
HPP	Orange Juice	Naringenin and Hesperetin	400 MPa, 40 °C, 1 min	↑ HP treatment led to increased naringenin (20.16%) and hesperetin (39.88%) contents	[107]
HPP	Orange Juice	Naringenin and Hesperetin	400 mPa, 40 °C, 1 min	↑ Increase in total flavanone content extracted (15.46%). Losses during the storage	[108]
HPP	Mandarin Juice	Total flavones and individual (Vicenin-2, apigenin d); Total flavanones and individual (naringin-d, naritutin, hesperidin, dydimin)	150 MPa	Compared to fresh juice, processing had a positive effect on the bio-accessibility of flavonoids, although pasteurisation provided better results	[109]
PEF	Orange Juice	TPC	100 µs, 30 kV/cm	↓ Treated juice with PEF had a decrease in TPC (T = 4 ± 2 °C, for 7 weeks): 1.045 mg/mL compared to pasteurised juice (1.070 mg/mL); (T = 10 ± 2 °C, for 6 weeks): 0.941 mg/mL compared to pasteurised juice (0.985 mg/mL)	[94]
PEF	Orange, pomelo and lemon juice	TPC	70 µs, 3 kV/cm	↑ The PEF treatment increased the yield of juice (after pressing) by 25% for orange, 37% for pomelo, and 59% for lemon	[86]
PEF	Orange Juice	Naringenin and Hesperetin	750 µs, 35 kV/cm	PEF juice did not show significant changes in flavanone content with regard to freshly squeezed orange juice. Losses during the storage.	[108]
PEF	Orange Juice	Naringenin and Hesperetin	750 µs, 35 kV/cm	PEF treatment did not modify flavanone content.	[107]

**Table 4:** Effects of innovative processing technologies on citrus vitamin C content.

IPT	Application Matrix	Detected Compounds	Mechanisms Involved	Results	Ref.
MH	Orange juice	Vitamin C	455 W, 180 s, uncontrolled temperature	↓ Degradation of vitamin C during MH compared to heat treatment	[112]
MH	Grapefruit juice	Vitamin C	900 W, 30 s	No significant differences between MH and heat treatment	[104]
MH	Orange Juice	Vitamin C	900 W, 30 s	No significant differences between MH and CH	[113]
US	Lime Juice	Vitamin C	25 kHz, 20 °C	↑ Increase in ascorbic acid content was observed compared to heat treatment	[105]
US	Grapefruit juice	Vitamin C	28 kHz, 20 °C	↑ With sonication treatment for 90 min an increase was observed (35.75 mg/ 100 mL) compared to control 0 min (27.83 mg/100 mL)	[106]
HPP	Orange Juice	Vitamin C	500 MPa, 35 °C, 5 min	↑ Better retention of ascorbic acid compared to heat treatment	[114]
HPP	Orange Juice	L-ascorbic acid and total Vitamin C	400 MPa, 40 °C, 1 min	↓ Treatments caused a significant decrease in L-AA content (7.79%)t Total Vitamin C did not exhibit any change	[107]
HPP	Orange Juice	Vitamin C	4000 bars, 5 min	↑ Treated juice with HPP had a good result: (T = 4 ± 2 °C, for 7 weeks): 42.59 mg/100 mL compared to pasteurised juice (35.58 mg/100 mL); (T = 10 ± 2 °C, for 6 weeks): 42.98 mg/100 mL compared to pasteurised juice (18.74 mg/100 mL)	[94]
PEF	Orange Juice	L-ascorbic acid and total Vitamin C	750 µs, 35 kV/cm	↓Treatments caused a significant decrease in L-AA content (7.79%) and in total Vitamin C (8.24%)	[107]
PEF	Orange Juice	Vitamin C	100 µs, 30 kV/cm	↑ Treated juice with PEF had a good result: (T = 4 ± 2 °C, for 7 weeks): 42.66 mg/100 mL compared to pasteurised juice (35.58 mg/100 mL); (T = 10 ± 2 °C, for 6 weeks): 43.03 mg/100 mL compared to pasteurised juice (18.74 mg/100 mL)	[94]

**Table 5:** Effects of innovative processing technologies on carotenoids content present in citrus juice.

IPT	Application Matrix	Detected Compounds	Mechanisms Involved	Results	Ref.
OH	Grapefruit juice and Blood orange Juice	cis-violaxanthin, lutein, zeaxanthin, b-cryptoxanthin, lycopene, and β-carotene	5 kW, 50 Hz, 0.1–3 kV·m^−1^	↓ 40–70% for xanthophylls with conventional heating; ↓ was observed using OH heating: 30% for epoxyxanthophylls and 20% for hydroxyxanthophylls	[119]
MH	Orange Juice	Violaxanthin, antheraxanthin, lutein, zeaxanthin, b-cryptoxanthin, α-carotene, and β-carotene	3 kW, 2.45 GHz	↓ At 85 °C, a decrease of approximately 50% was observed for almost all carotenoids after 1 min of MW heating	[118]
HPP	Orange Juice	Lutein, zeaxantin, α-cryptoxanthin, β-cryptoxanthin, α-carotene, β-carotene	400 MPa, 40 °C, 1 min	↑ HPP treatment led to a carotenoid release (53.88%)	[107]
HPP	Grapefruit juice	All-trans-lycopene and β-carotene	402 ± 1.9 MPa, 31.8 ± 0.5 °C, 3 min	↑ HPP showed better results in carotenoids recovery, 14 days after storage, compared with untreated juice and thermal treated juice	[120]
US	Mandarin juice	Total carotenoids (β-carotene equivalents)	50 °C, 750 W; 36 min	↑ Total carotenoid content was higher than in the untreated samples due to US cavitation effects.	[121]
HPP	Orange Juice	Total carotenoids	4000 bars for 5 min	↑ Treated juice with HHP had a good result: (T = 4 ± 2 °C, for 7 weeks): 997.2 µg/100 g compared to pasteurised juice (913.3 µg/100 g); (T = 10 ± 2 °C, for 6 weeks): 1087.4 µg/100 g compared to pasteurised juice (854.2 µg/100 g)	[94]
HPP	Valencia Orange Juice	Lutein, zeaxantin, α-cryptoxanthin, β-cryptoxanthin, α-carotene, β-carotene	400 mPa, 40 °C, 1 min	↑ Increase in the extractability of each individual carotenoid with regard to untreated juice and in total carotenoid content (45.19%). Furthermore, good results during storage (4 °C) were observed	[108]
PEF	Valencia Orange Juice	Lutein, zeaxantin, α-cryptoxanthin, β-cryptoxanthin, α-carotene, β-carotene	750 µs, 35 kV/cm	PEF juices did not exert any change on carotenoid content value in comparison with freshly squeezed orange juice. A good result during storage (4 °C) was observed	[108]
PEF	Orange Juice	Lutein, zeaxantin, α-cryptoxanthin, β-cryptoxanthin, α-carotene, β-carotene	750 µs, 35 kV/cm	PEF treatment did not modify individual or total carotenoid content	[107]
PEF	Orange Juice	Total carotenoids	100 µs, 30 kV/cm	↑ Treated juice with PEF had good results: (T = 4 ± 2 °C, for 7 weeks): 964.2 µg/100 g compared to pasteurised juice (913.3 µg/100 g); (T = 10 ± 2 °C, for 6 weeks): 1107.8 µg/100 g compared to pasteurised juice (854.2 µg/100 g)	[94]

**Table 6:** Impact of innovative processing technologies on microbiological contamination in citrus juice.

IPT	Application Matrix	Mechanisms Involved	Results	Ref.
PEF	Orange juice	35 KV/cm, 4 μs pulse width, 32.5 °C	5.1-log reduction in *S. cerevisiae*	[125]
PEF	Grapefruit juice	1 kHz, 600 μs, 25 kV/cm, at 40 °C	1.72-log reduction in total psychrophiles and 1.66-log reduction in yeasts and moulds	[128]
PEF	Orange juice	22 and 20 kV/cm at 45 and 55 °C	2.22-log reduction in *E. coli*, *S. typhimurium,* and non-pathogenic microbes	[129]
PEF	Orange juice	16,3 KV/cm, 20 μs pulse width, 100 °C	4-log reduction in *B. subtilis*	[130]
HPP	Orange juice	200 MPa, 15 min; 300 MPa 15 min, at 22 °C	5-log reduction in *E. coli*; 5-log reduction in *Lb. plantarum*	[131]
HPP	Grapefruit juice	250 MPa, at 60 °C for 3 min	2.5-log reduction in total psychrophiles and 1.1-log reduction in yeasts and moulds	[132]
HPP	Orange juice	600 MPa/1 min, 17 °C	Complete inactivation of total psychrophiles, *Enterobacteriaceae*, *E. coli*, lactic acid bacteria, yeasts, and moulds	[133]
US	Orange juice	24 kHz, 1, 10, 20, and 30 min, 43 to 45 °C	1.6-log reduction in total psychrophiles and 0.9-log reduction in yeasts and moulds	[134]
US	Orange juice	20 kHz, Varying amplitude levels (0.4, 7.5, 37.5 μm), 15 min, below 30 °C	5.9-log reduction in *E. coli*	[135]
US	Orange juice	500 kHz, 240 W for 15 min, 20 °C	0.1- to 1.08-log reduction in total mesophilic bacteria and yeasts and moulds	[127]
